# Commentary: The Dark Side of Top Level Sport: An Autobiographic Study of Depressive Experiences in Elite Sport Performers

**DOI:** 10.3389/fpsyg.2016.01588

**Published:** 2016-10-24

**Authors:** Jonathan Males

**Affiliations:** Performance1 LtdLondon, UK

**Keywords:** depression, performance, qualitative methods, sport psychology, elite athletes

This intriguing article explored a challenging topic using a novel albeit somewhat controversial methodology. This commentary is made from the perspective of an applied sports psychology practitioner who has conducted qualitative research, with the primary focus on how this informs best practice in consulting with athletes.

Newman et al. (2016) certainly raised questions for practitioners about the use of autobiographies, particularly those which have had input from a second author. The act of writing encourages retrospective sense making (Gersie and King, 2003). A skilled writer creates a narrative coherence that is engaging for the reader. This is challenging when they are primarily used as data sets for qualitative enquiry. As Newman et al. (2016) suggest, stories of depression are marketable, and does this perhaps mean that authors or ghostwriters are prone to exaggeration? One should also be curious about how well the narrative description of depression matched the lived experience of the athletes at the time. Were the athletes able to describe their situation using eloquent metaphors or alliteration in the midst of their despair? Practitioner experience would question whether a depressed athlete is likely to have such fluent self-awareness—the reality of depression is more likely to be informed by a fuzzy logic, which is harder to articulate and marked more by withdrawn silences or frustrated outbursts than eloquent prose.

The authors chose to focus on elite athletes, and by definition any athlete who writes an autobiography is likely to have, or had, a high public profile. To what extent is being in the public eye a mediating factor in the role of depression and performance? Certainly it would seem to add weight to both the approval of others and the emotional cost of failure, key factors that emerged in the analysis. This train of thought raises the possibility that one should consider celebrity performers in other fields such as music or acting who have reported depression. A quick Wikipedia search suggests there is no shortage of candidates for such a study. Do they report a similar relationship between their “craft” and depression as the elite athletes in this study? From my own limited reading it seems likely they do (e.g., Clapton, 2007). If so, then perhaps the issue is less about depression and elite sport performance and more about being human and the search for a meaningful identity.

As a practitioner, I have encountered a small handful of “non-celebrity” yet elite athletes with depression and similar causal factors were present to those identified in this study; especially an over-reliance on sporting success as a source of self-worth, identity foreclosure and conflict over whether to continue with their sport. Overall I was able to recognize the authors' analysis of the factors likely to lead to, or result from, depression. The warning signs of “an overly self-critical nature, perfectionism, and fear of failure” are usefully defined.

A further issue is raised by Newman et al. (2016) which is the ethical boundary as an applied sport psychology practitioner when encountering a mental health issue such as depression. Assuming that a strong working relationship has been established, sport psychologists may find themselves a confidante and source of emotional support for an athlete suffering from depression. Yet I have heard many practitioners in sport psychology express doubts about their competence to deal with mental health problems, indicating a reluctance to get too “close” and preferring to refer to a clinical psychologist. Given that the major risk factor with depression is self-harm and in the worst-case scenario suicide, caution is justified. I also believe that this highlights the need for sport psychologists to be trained in counseling skills and to possess sufficient personal integrity to be able to “hold” an effective relationship with a client in psychological distress. Depression is a complex and multi-faceted condition that often requires both medical and psychological intervention.

Whilst depression is of one of the most common mental health problems, others such as anxiety and eating disorders are prevalent too. An example of an effective and pragmatic educational Mental Health program for coaches and athletes comes from the Australian Institute of Sport. Sebbens et al. (2016) developed and tested a 4 h workshop that identifies and normalizes mental health problems and gives a simple framework, “reach out, refer and remain supportive” that is just as applicable to non-clinically trained sport psychologists as it is to coaches or parents.

My closing reflection is on the quandary that practitioners can face when working with a depressed elite performer. Is the practitioner's role to enable the athlete to perform, or to help them live a healthy and satisfying life? Whilst these two outcomes are not necessarily exclusive, in many elite sport settings the explicit expectation from coaches and management will be to prioritize performance. So whilst a focus on the athlete's internal experience and psychological dynamics is relevant, let's not forget the impact of systemic and cultural factors on athlete's mental health.

## Author contributions

The author confirms being the sole contributor of this work and approved it for publication.

### Conflict of interest statement

The author declares that the research was conducted in the absence of any commercial or financial relationships that could be construed as a potential conflict of interest.
